# Microbial Natural Product Alternariol 5-*O*-Methyl Ether Inhibits HIV-1 Integration by Blocking Nuclear Import of the Pre-Integration Complex

**DOI:** 10.3390/v9050105

**Published:** 2017-05-10

**Authors:** Jiwei Ding, Jianyuan Zhao, Zhijun Yang, Ling Ma, Zeyun Mi, Yanbing Wu, Jiamei Guo, Jinmin Zhou, Xiaoyu Li, Ying Guo, Zonggen Peng, Tao Wei, Haisheng Yu, Liguo Zhang, Mei Ge, Shan Cen

**Affiliations:** 1 Institute of Medicinal Biotechnology, Chinese Academy of Medical Sciences and Peking Union Medical School, Beijing 100050, China; jiweiding1223@aliyun.com (J.D.); zjyuan815@163.com (J.Z.); maling26@163.com (L.M.); mizeyun@126.com (Z.M.); wyblily@sina.com (Y.W.); zhou_jim@hotmail.com (J.Z.); xiaoyulik@hotmail.com (X.L.); pumcpzg@126.com (Z.P.); 2 School of Pharmacy, Shanghai Jiaotong University, Shanghai 200040, China; yangzjun80@sina.com (Z.Y.); hccbred@gmail.com (M.G.); 3 Institute of Materia Medica, Chinese Academy of Medical Sciences and Peking Union Medical School, Beijing 100050, China; guojiamei@imm.ac.cn (J.G.); yingguo6@imm.ac.cn (Y.G.); 4 Department of Food Science, Beijing Union University, Beijing 100101, China; weitao@buu.edu.cn; 5 Institute of Biophysics, Chinese Academy of Sciences, Beijing 100101, China; yuhaisheng@moon.ibp.ac.cn (H.Y.); zhanglgf@hotmail.com (L.Z.)

**Keywords:** HIV integration, pre-integration complex, alternariol 5-*O*-methyl ether, nuclear import, natural product

## Abstract

While Highly Active Antiretroviral Therapy (HAART) has significantly decreased the mortality of human immunodeficiency virus (HIV)-infected patients, emerging drug resistance to approved HIV-1 integrase inhibitors highlights the need to develop new antivirals with novel mechanisms of action. In this study, we screened a library of microbial natural compounds from endophytic fungus *Colletotrichum* sp. and identified alternariol 5-*O*-methyl ether (AME) as a compound that inhibits HIV-1 pre-integration steps. Time-of addition analysis, quantitative real-time PCR, confocal microscopy, and WT viral replication assay were used to elucidate the mechanism. As opposed to the approved integrase inhibitor Raltegravir, AME reduced both the integrated viral DNA and the 2-long terminal repeat (2-LTR) circular DNA, which suggests that AME impairs the nuclear import of viral DNA. Further confocal microscopy studies showed that AME specifically blocks the nuclear import of HIV-1 integrase and pre-integration complex without any adverse effects on the importin α/β and importin β-mediated nuclear import pathway in general. Importantly, AME inhibited Raltegravir-resistant HIV-1 strains and exhibited a broad anti-HIV-1 activity in diverse cell lines. These data collectively demonstrate the potential of AME for further development into a new HIV inhibitor, and suggest the utility of viral DNA nuclear import as a target for anti-HIV drug discovery.

## 1. Introduction

An essential step in the replication cycle of human immunodeficiency virus (HIV)-1 is integration of viral DNA into host chromosome DNA, which is catalyzed by viral integrase (IN). Since there are no cellular homologs in human cells and the reactions which INs catalyze are unique, they avoid off-target effects and adverse drug actions. Additionally, IN is necessary for HIV-1 replication and several key residue mutations render it inactive, thus, it represents an ideal target for the discovery of HIV-1 inhibitors [1]. HIV integration includes three essential steps: 3′-processing, nuclear import of pre-integration complex (PIC), and strand transfer. Early IN inhibitor (INI) design and discovery have focused on directly inhibiting IN enzymatic activities. The β-Diketo acid derivatives (DKAs) are the first compounds that were shown to specifically impede strand transfer [2]. DKAs inspired the development of the first-generation INIs including Raltegravir (RAL) and Elvitegravir (EVG), which are also referred to as integrase strand transfer inhibitors (INSTIs) [3]. These INSTIs show a low genetic barrier to resistance development and IN resistance mutations show cross-resistance to RAL and EVG [4]. The second-generation INSTIs including MK2048 and dolutegravir (DTG) inhibit most RAL-resistant HIV-1 mutants. However, development of cross-resistant viral strains appears to be inevitable because both the first and second generations of INSTIs share an overlapping binding site in the IN catalytic domain. Indeed, DTG resistance mutations including G118R and R263K were recently identified, and some of them conferred cross-resistance to RAL and EVG [5]. Development of allosteric IN inhibitors (ALLINIs) provides an alternative approach to discover compounds that are effective against INSTI-resistant HIV-1 [6]. Yet, their effectiveness in clinics awaits further studies.

In addition to targeting the catalytic activity of HIV-1 IN, nuclear entry of HIV-1 pre-integration complex (PIC) represents a promising target. PIC is generated in the cytoplasm following the reverse transcription of viral RNA into viral DNA [7]. The viral components include IN, nucleocapsid (CA), matrix (MA), viral protein R (Vpr), and reverse transcriptase (RT) [8]. Several host proteins including lens epithelium-derived growth factor (LEDGF/p75), barrier-to-autointegration factor (BAF), and integrase interactor 1 have also been found in PIC [9]. Transport of PIC into the nucleus is the prerequisite for the integration of HIV-1 DNA into cellular chromosomes [7]. Yet, it is still uncertain which nuclear import pathway is adopted by PIC. Several lines of evidence support the involvement of importin α/β [10,11,12,13]. Other studies suggest a key role of a non-classical nuclear import localization signals (NLS) of IN in the nuclear accumulation of PIC [14,15]. Mutations in MA and CA also block PIC nuclear transport, suggesting that MA and CA also promote the nuclear import of PIC [16,17,18]. In addition to these viral proteins, cellular import factors are also believed to assist PIC nuclear import. For example, nuclear pore complex component nucleoporin 153 (Nup153) and transportin-3 (TNPO3) operate synergistically in nuclear import of PIC [19,20]. Importin α3 (Imp α3), importin 7 (Imp7), and transportin-SR2 (TRN-SR2) have been shown to interact with HIV-1 IN and affect viral nuclear import [13,21,22,23].

Although the detailed mechanisms and the nature of host/viral factors behind nuclear import of HIV-1 PIC are still being investigated, some efforts have been made to find small compounds that can target this process. For example, styrylquinoline derivatives show post-entry, pre-integration antiviral activities [24]. An importin α/β pathway inhibitor called ivermectin inhibits IN nuclear transport and HIV-1 replication [25]. Mifepristone was reported to specifically block nuclear import of HIV-1 IN [26]. These studies support the idea that inhibiting IN nuclear import is a feasible approach for discovering new HIV-1 inhibitors. In the present study, we have identified alternariol 5-*O*-methyl ether (AME), a natural product from endophytic fungus *Colletotrichum* sp, as a novel anti-HIV compound. Further studies revealed that AME specifically blocks nuclear import of HIV-1 PIC and does not affect the importin α/β and importin β-mediated nuclear import pathway in general. Importantly, AME inhibits both wild-type HIV-1 and RAL-resistant viruses, suggesting the potential of AME as a prototype of IN nuclear import inhibitors with the potential to be further developed into a new generation of anti-HIV compounds.

## 2. Materials and Methods

### 2.1. Cell Culture and Transfection

HEK293T cells and HeLa cells were maintained in Dulbecco’s modified Eagle’s Medium supplemented with 10% Fetal Bovine Serum (FBS). MT-2, MT-4, SupT1, and Jurkat cells were maintained in RPMI1640 medium supplemented with 10% FBS. Peripheral blood mononuclear cells (PBMC) from healthy donors were isolated by Ficoll-Hypaque centrifugation and incubated in RPMI1640 medium containing 5 µg/mL phytohemagglutinin (PHA) and 50 U/mL human recombinant IL-2 for 72 h prior to anti-viral assays. A total of 1 × 10^6^ HEK293T cells were transfected with 0.6 µg pNL4-3luc.R-E- in the presence of 60 µM AME or DMSO using Lipofectamine2000 (Invitrogen, Carlsbad, CA, USA). Two days post-transfection, cells were harvested with RIPA buffer (50 mM Tris-HCl (pH 7.4), 150 mM NaCl, 1% NP-40, 0.1% SDS) and the levels of Gag in transfected cells were assessed by Western blotting. For immunostaining assay, 0.1 µg IN-EGFP or Rev-HA was transfected into 2 × 10^4^ HEK293T cells using Lipofectamine2000 in NuncLab-TeklI (Thermo Fisher Scientific, Pittsburgh, PA, USA).

### 2.2. Plasmids and Reagents

The HIV pNL4-3.Luc.R-E- vector contains a full-length HIV-1 proviral DNA in which *env* is defective and *nef* was replaced by luciferase. The vesicular stomatitis virus glycoprotein (VSV-G) expressing vector pHIT/G was provided by Johnny He [15]. To construct IN-EGFP, the coding region of HIV-1 IN was amplified by PCR and inserted into the pEGFP-C1 expression vector (Clontech Laboratories, Palo Alto, CA, USA) at EcoR I and Bgl II. The primers used to amplify the HIV-1 IN are as follows: 5′TAG GAA TTC ATG TTT TTA GAT GGA ATA GAT AAG 3′ (sense) and 5′TAG GGA TCC ATC CTC AT C CTG TCT ACT TGC CAC 3′ (antisense). RAL-resistant mutant V151L was kindly provided by Yong Xiao (McGill University, Montreal, QC, Canada). Another three Q148 pathway mutants G140SQ148H, Q148H, and Q148S, were kindly provided Matthew D. Marsden (University of California, Los Angeles, CA, USA) [27]. To construct subtype C founder/transmitted and chronic infection Luciferase Reporter pseudovirus, the fragment from 5′LTR to the initiation site of env CDS of pNL4-3.Luc.R-E- was replaced by the counterpart of pZM247Fv1 (NIH AIDS REAGENT PROGRAM Catalog #11941) or pIndie-C1 [28] using an IN-Fusion HD cloning kit (Clontech), these chimera constructs were named pZM247Fv1Luc [29] (founder/transmitted reporter virus) and pIndie-C1-Luc (chronic infection virus), respectively.

### 2.3. Screening for Anti-HIV Compounds from a Library of Microbial Natural Compounds

Microbial natural products were obtained from fungus *Colletotrichum* sp. Briefly, the fermented substrate was extracted with AcOEt and evaporated to get crude extract. Then, the crude extract was fractionated by silica gel and purified by semi-preparation RP-HPLC to obtain the compounds, including AME. The screening was performed as previously described [30]. Briefly, 2 × 10^5^ HEK293T cells were co-transfected with 0.6 µg of pNL4-3Luc.R-E- and 0.4 µg of pHIT/G. After 48 h, the VSV-G pseudotyped HIV-1 viruses were harvested by filtration through a 0.45 mm filter and the concentration of viral capsid protein was determined by p24 antigen capture ELISA. A total of 1 × 10^5^ SupT1 cells were subject to VSV-G pseudotyped HIV-1 infection (MOI = 1) in the absence or presence of test compounds (Efavirenz used as positive control). The inhibition rate was determined by a firefly Luciferase Assay System (Promega, Madison, WI, USA) at 48 h post-infection.

### 2.4. Assay for Measuring the Inhibitory Activity of Compounds on Different HIV-1 Strains

The inhibitory activity of AME on infection by a typical HIV-1 strain, NL4-3luc.R-E-, and three RAL-resistant strains, G140SQ148H, Q148H, and Q148S, were tested in SupT1 cells. Briefly, 1 × 10^5^ cells were infected by VSV-G-pseudotyped HIV-1 viruses, followed by addition of compounds at serial dilutions. After further incubation at 37 °C for 48 h, cells were harvested and luciferase activities were measured by 960 luminometer. The concentration of the compound for inhibiting 50% viral replication (IC50) was determined by Origin 8.0 software.

### 2.5. Cytotoxicity Assay

AME was added to HEK293T cells at 1 × 10^5^ per well, followed by incubation at 37 °C for 48 h. Ten microliters of CCK-8 reagent were added to the cells. After incubation at 37 °C for 4 h to allow color development of the XTT formazan product, the absorbance of each well was read at 450 nm. The 50% cytotoxicity concentration (CC50) was generated by Origin 8.0 software.

### 2.6. Time of Addition Experiment

A total of 1 × 10^5^ SupT1 cells/well were infected with VSV-G-pseudotyped NL4-3Luc.R-E- viruses and incubated at 4 °C for 1 h in order to synchronize infection. The unbound viruses were washed off with phosphate-buffered saline (PBS) and then the cells were incubated at 37 °C. Four nM efavirenz (EFV), 25 nM RAL, 76 nM 3TC, and 60 µM AME were added at 0, 0.5, 1, 2, 4, 6, 7, 12, 15, 18, and 21 h. Two days post-infection, cells were lysed with cell culture lysis buffer (Promega), luciferase activities were measured, and the time-response curves were generated using Origin 8.0 software.

### 2.7. Semi-Quantitative Real-Time PCR

A total of 5 × 10^6^ SupT1 cells were infected with VSV-G pseudotyped NL4-3Luc.R-E- viruses in the treatment of 4 nM EFV, 25 nM RAL, or 60 µM AME. Cells were harvested at 3, 8, or 24 h post-infection. After washing with PBS, total DNA was extracted using DNeasy Blood&Tissue Kit (Qiagen, Hilden, Germany). An aliquot of each sample was analyzed by PCR. The PCR program was a relative quantitative procedure in an Mx3000P real time PCR system (Agilent Technologies Inc., Palo Alto, CA, USA). The primers used in the PCR were: U5-Gag sense, 5′TGT GTG CCC GTC TGT TGT GTG A3′; U5-Gag antisense, 5′TCA GCA AGC CGA GTC CTG CGT3′; Alu-LTR sense, 5′TCC CAG CTA CTC GGG AGG CTG AGG3′; Alu-LTR antisense, 5′AGG CAA GCT TTA TTG AGG CTT AGC3′; 2-long terminal repeat (2-LTR) sense, 5′AAC TAG GGA ACC CAC TGC TTA AG3′; and 2-LTR antisense, 5′ TCC ACA GAT CAA GGA TAT CTT GTC 3′. Total viral DNA, integrated DNA, and 2-LTR were expressed as copy numbers per cell, with DNA template normalized by the GAPDH gene that was amplified using GAPDH primers.

### 2.8. Immunostaining

HeLa cells were grown on coverslips in the treatment of DMSO, 25 nM RAL, 60 µM AME, or 25 µM ivermectin for 48 h, and then fixed in 4% paraformaldehyde and permeabilized in 0.4% TritonX-100 followed by incubation in primary and secondary antibodies. The primary antibody was anti-IN at 1:500 (Abcam, Cambridge, MA, USA) [31]. The secondary antibody was TRITC-conjugated goat anti-mouse immunoglobulin (Ig)G or FITC-conjugated goat anti-mouse IgG (Sigma-Aldrich, St Louis, MO, USA). Fluorescence images were acquired on an Olympus FV1000 confocal fluorescence microscope.

### 2.9. Nuclear and Cytoplasmic Fractionation

HEK293T cell cytosol and nucleus were prepared according to the manual of the Nuclear and Cytoplasmic Protein Extraction Kit (Sangon Biotech, Shanghai, China). In brief, 1 × 10^6^ HEK293T cells were collected by centrifugation at 3000 rpm at 4 °C. Cells were washed twice with PBS and once with Solution A supplemented with protease inhibitors, DTT, phosphatase inhibitors and PMSF. After sonication, cell lysate were centrifuged at 12,000 rpm for 30 min at 4 °C. The supernatant contained cell cytosol. The pellet was incubated with Solution B supplemented with protease inhibitors, DTT, phosphatase inhibitors and PMSF, and then centrifuged at 12,000 rpm for 30 min at 4 °C; the supernatant contained cell nuclei. Each sample was added with 5× SDS lysis buffer and boiled, then detected by SDS-PAGE and Western blotting. The PVDF membranes were immunoblotted with mouse anti-IN, anti-β-actin, and anti-MCM2 antibodies (Abcam) and then with complementary horseradish peroxidase (HRP)-conjugated secondary antibodies.

### 2.10. Viral Preparation and Infection Assay

HIV-Luc Reporter viruses were produced from HEK293T cells by transfecting with VSV-G and pNL4-3luc.R-E-. Founder/transmitted Reporter viruses were produced by transfecting with VSV-G and pZM247Fv1Luc or pIndie-C1-Luc as a chronic infection control. The supernatants were filtered and stored at −80 °C for future use. For single round infection assays, 5 × 10^5^/mL SupT1 cells were infected with pseudotyped viruses. Two days post-infection, cells were lysed with cell culture lysis buffer (Promega) for 20 min at 37 °C, 6 µL of the lysate was added with 40 µL substrate, luciferase activities were measured by 960 luminometer.

For detection of viral entry, 1 × 10^4^ SupT1 cells per well were seeded in 96-well plates and divided into two populations. In one population, the cells were treated with T-20, AME, or DMSO as vehicle control at the concentrations of 2 fold IC_50_, immediately followed by infection with NL4-3Luc pseudoviruses. In another population, cells were incubated with pseudoviruses at 4 °C for 1 h and then the cells were washed with PBS followed by treatment of T-20, AME or DMSO. Forty-eight h later, cells were harvested and Luc activity was measured. Luc activity of each sample was normalized by the vehicle control.

For WT HIV infection, HIV-1 NL4-3 viruses were produced from HEK293T cells by transfecting with pNL4-3. One million PBMC were seeded in each well in a 96-well plate and incubated with DMSO, 2 nM EFV, 35 µM or 180 µM AME for 5h. Then, the medium was refreshed and cultured for a further 72 h. Supernatants were harvested at day 3 and day 5 and assayed for HIV-1 p24 content by an enzyme-linked immunosorbent assay (ELISA, ZeptoMetrix Corp., Buffalo, NY, USA).

### 2.11. In Vitro Integrase Assay

The wells of a microtiter plate were coated with a 30 base pair (bp) stretch of the sequence encoding U5-LTR. HIV-1 integrase and tested compounds were added and incubated in reaction buffer (25 mM MOPS, pH 7.2, 20 mM Tris-HCl, pH 7.8, 20 mM DTT, 15 mM MnCl_2_ and 0.2%TritonX-100) at 37 °C for 60 min. Then, a 20 bp oligonucleotide of 3’-biotin-conjugated target substrate was added into the microplate and incubated for another 60 min. After washing with PBS, the microplate was added with horseradish peroxidase (HRP) dilution buffer (10 mM Tris-HCl, pH7.5, 0.15M NaCl, 1 mM EDTA, 1%BSA) and incubated at 37 °C for 30 min. Then, the plate was washed with PBS. One hundred µL of TMB buffer (3,3′,5,5′-tetramethylbenzidine) was dispensed into each well, incubated for 15 min in the dark, and an equal volume of stopping solution (2M H_2_SO_4_) was added. The optical density was read at 450 nm.

## 3. Results

### 3.1. AME Inhibits HIV Infection

**Figure 1:**
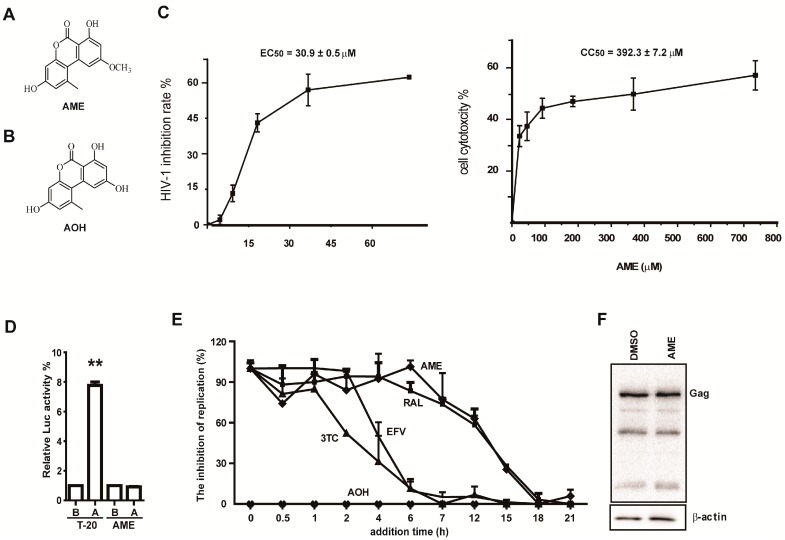
Alternariol 5-*O*-methyl ether (AME) potentially inhibits early stage of human immunodeficiency virus (HIV)-1 replication. (**A**) The structure of AME was solved through spectroscopic analysis; (**B**) The structure of alternariol (AOH); (**C**) SupT1 cells were infected with HIV-1 NL4-3luc.R-E- pseudoviruses in the presence of different concentrations of AME. 48 h post-infection, cells were lysed and luciferase activity was measured. A dose-response curve was generated using the Origin 8.0 software; (**D**) SupT1 cells were infected with HIV-1 NL4-3luc.R-E- pseudoviruses before or after treatment with either AME or T-20 at 2 fold IC50. Luciferase activity was measured before (B) or after (A) viral infection. Relative luciferase activity was a ratio of the luciferase activity in cells infected before AME (or T20) treatment over that in cells infected after drug treatment (set as 1); (**E**) A time of addition (TOA) experiment was carried out to determine the step of HIV-1 infection that AME inhibits. Briefly, after infection of SupT1 cells with HIV-1 NL4-3luc.R-E- pseudoviruses, inhibitors of the indicated concentrations were added at different time points ranging from 1 to 24 h post-infection. The relative inhibition rate was calculated by dividing the inhibition rate at 0 h for each compound. White diamond: AME; black diamond: efavirenz (EFV); triangle: 3TC; rectangular: Raltegravir (RAL); cross: AOH. Viral infection was determined by measuring luciferase activity at 48 h post-infection. As controls, reverse transcriptase (RT) inhibitors EFV and 3TC, integrase strand transfer inhibitors (INSTIs) RAL, as well as inactive congener AOH were also tested; (**F**) HEK293T cells were transfected with pNL4-3luc.R-E-. Forty-eight h post-transfection, cells were lysed, and Gag expression was determined by Western blotting. Data represent the mean ± SD of three independent experiments.

In an effort to discover new anti-HIV compounds, we examined a library of microbial natural products as previously described [30]. The results revealed a number of hits including alternariols from endophytic fungus *Colletotrichum* sp., among which AME showed antiviral activity (Figure 1A). In contrast, its derivative alternariol (AOH) (Figure 1B) had little effect on HIV-1 infection with an inhibition rate ~6% even at a concentration of 300 µM. AME inhibited the HIV-1 infection with an EC_50_ of 30.9 ± 0.5 µM (*n* = 3) in a one-cycle HIV-1 infection assay (Figure 1C), which is much lower than a CC_50_ of 392.3 ± 7.2 µM (*n* = 3). The result excludes the possibility that the anti-HIV activity was due to the cytotoxicity of AME.

To explore the anti-HIV mechanism of AME, we first examined whether AME impairs viral entry. T-20, a synthetic peptide which blocks viral entry, was used as positive control. As shown in Figure 1D, the antiviral effect of HIV-1 entry inhibitor T-20 was significantly reduced when the compound was added at post-infection, while no difference in the inhibitory effect of AME was observed regardless of the addition of the compound before or after viral infection. This indicates that AME acts, at least in part, downstream of the viral entry step. Next, we further conducted the time-of-addition experiment (TOA), which has been used to pinpoint which step of HIV-1 replication cycle is blocked by antiretroviral compounds. Briefly, AME was added at the different time points after infection of SupT1 cells with pseudotyped HIV-1 NL4-3luc.R-E-, and 48 h after infection, luciferase activities were measured to determine the anti-HIV effect of AME. As controls, nucleoside reverse transcription inhibitor Lamivudine (3TC), non-nucleoside reverse transcription inhibitor, efavirenz (EFV), integrase inhibitor RAL, and inactive congener AOH were used to treat infected SupT1 cells. 3TC, EFV, and RAL exhibited their antiviral activities within 1 h, 2 h, and 7 h post-infection, respectively, which reflect the time required for completion of reverse transcription and integration. AME still inhibited HIV-1 when added 7 h after infection, and lost its inhibitory activity when added 15 h after infection, which corresponds to the time-activity curve of RAL (Figure 1E). Thus, this suggests that AME likely inhibits HIV at a step in the replication cycle similar to that of RAL. As expected, AOH showed no anti-HIV activity at all the time points we tested. To further rule out the possibility that the reduced viral infectivity resulted from an inhibitory effect of AME on transcription or translation of the viral gene, HEK293T cells were transfected with pNL4-3luc.R-E- in the presence of 60 µM AME or DMSO. Levels of Gag in transfected cells were assessed by Western blotting. The results showed that AME had no effect on HIV-1 Gag expression (Figure 1F). Taken together, these data suggest that AME may inhibit HIV-1 replication by blocking viral integration.

### 3.2. AME Causes Defective HIV-1 Integration

**Figure 2:**
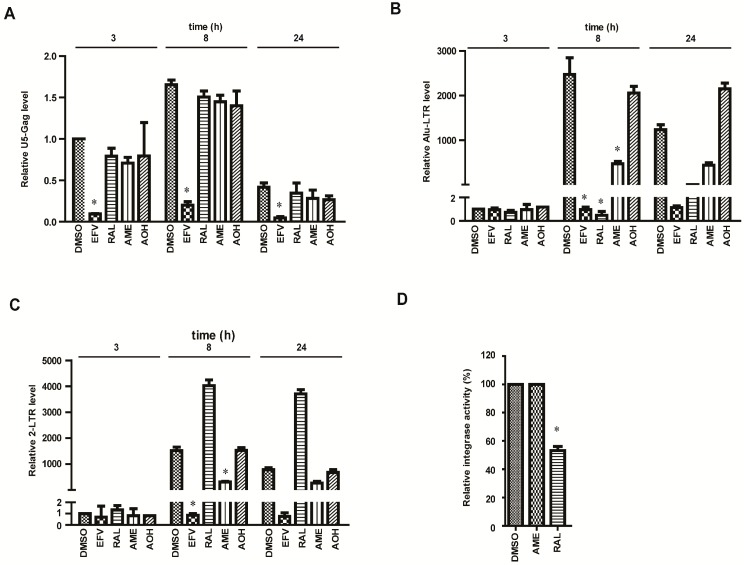
AME inhibits HIV integration. (**A**–**C**) SupT1 cells were infected with vesicular stomatitis virus glycoprotein (VSV-G) pseudotyped NL4-3Luc R-E- viruses with the treatment of DMSO, 4 nM EFV, 25 nM RAL, 60 µM AOH, or AME of indicated concentrations as described in the Materials and Methods section. DNA was extracted from infected cells at 3 h, 8 h, and 24 h post-infection. HIV early reverse transcription products (**A**), integrated DNA (**B**), and 2-LTR circles (**C**) were analyzed by RQ-PCR using the corresponding primers mentioned in the Materials and Methods section; (**D**) The activity of integrase was measured by an ELISA-based in vitro assay in the treatment of DMSO, 100 µM AME, and 100 nM RAL. * *p* < 0.05.

We next performed relative quantitative PCR (RQ-PCR) to directly assess the effect of AME on viral reverse transcription and integration. Primers were designed to detect the U5 and Gag (U5-Gag) sequences indicative of the following reverse transcription products: integrated viral DNA (Alu-LTR) and 2-LTR-containing DNA circles. A total of 1.5 × 10^6^ SupT1 cells per well were infected with pseudotyped HIV-1 NL4-3luc.R-E- in the treatment of DMSO, EFV, RAL, AOH, or AM. Cells were harvested at 3, 8, and 24 h after infection. DNA was extracted to quantify late reverse transcripts, 2-LTR circles, and integrated proviruses via qPCR. As shown in Figure 2A, among three compounds (EFV, RAL, and AME), only EFV caused a significant reduction in U5-Gag products, suggesting that AME had no effect on reverse transcription. Importantly, both RAL and AME severely reduced Alu-LTR DNA that represents viral integration product at 8 h post-infection (Figure 2B). Together with the results of the TOA analysis, we conclude that AME blocks the generation of integrated HIV-1 DNA.

2-LTR circles form at a low level in the nucleus through the action of cellular nonhomologous DNA end joining. Post nuclear entry blockage to HIV-1 infection is expected to result in an increase in 2-LTR circles. Therefore, the abundance of 2-LTR circles is an indirect measure for the nuclear import of the PIC. The decline of 2-LTR and subsequent block of proviral integration pinpoint a block of HIV replication between the late reverse transcription and the nuclear import steps. Indeed, the amount of 2-LTR circles was 3.9-fold higher in RAL-treated cells compared with DMSO-treated control cells at 8 h post-infection (Figure 2C). In contrast, AME reduced the level of 2-LTR circles. This suggests that, as opposed to RAL that impairs integration, AME appears to interfere with the nuclear import of HIV-1 DNA. In support of this result, AME did not affect 3′ processing and strand transfer reactions in vitro by ELISA at the concentration of 100 µM. (Figure 2D).

### 3.3. AME Blocks Nuclear Transport of PIC

**Figure 3:**
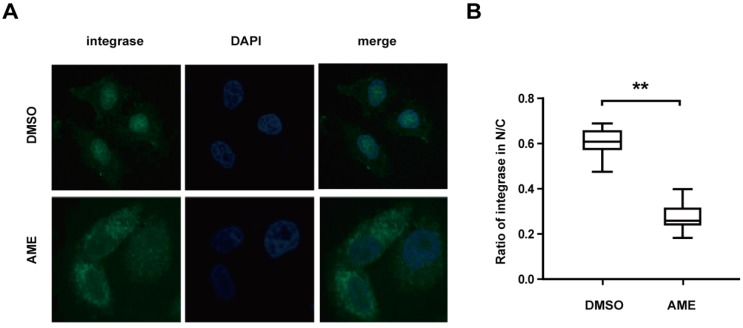
AME blocks pre-integration complex (PIC) nuclear import. (**A**) HeLa cells were infected with VSV-G pseudotyped NL4-3Luc R-E- viruses in the presence of 60 µM AME or DMSO. Twelve h post-infection, cells were fixed and labelled with mouse anti-integrase (anti-IN) antibody followed by FITC-conjugated goat anti-mouse secondary antibody, and the nuclei were stained with 4′6′-diamidino-2-phenylindole (DAPI). Cells were analyzed by confocal microscopy (with a 60× objective lens); (**B**) Statistical analysis for the ratio of integrase in nuclei and cell cytosol. Ratios of fluorescence in nucleus and whole cells at 488 nm were measured from 25 DMSO-treated cells and 25 AME-treated cells randomly selected from three independent experiments. The fluorescence density at 488 nm was quantified by ImageJ and statistical analysis was performed in Graphpad Prism 5.0 software. The data represent the percentage of PIC in the nucleus. Error bars represent standard deviation (SD) of all cells in each group. ** *p* < 0.01 (determined by Student’s *t* test).

Next, we measured the effect of AME upon nuclear import of HIV-1 PIC by performing an indirect immunofluorescence assay. HeLa cells were infected with HIV-1 in the presence of AME or DMSO. Subcellular localization of integrase was monitored by an indirect immunofluorescence assay using anti-integrase monoclonal antibody. IN was detected in the nucleus in DMSO-treated cells as opposed to the dominant cytoplasmic presence of IN in AME-treated cells (Figure 3A). When the ratio of nuclear fluorescence and total cellular fluorescence were measured, which represents the relative efficiency of IN nuclear import, 30% of IN was found in the nucleus of AME-treated cells as compared to about 60% of IN in the nucleus of DMSO-treated cells (Figure 3B). We, therefore, conclude that AME inhibits nuclear transport of HIV-1 IN that is an integral component of viral PIC. 

**Figure 4:**
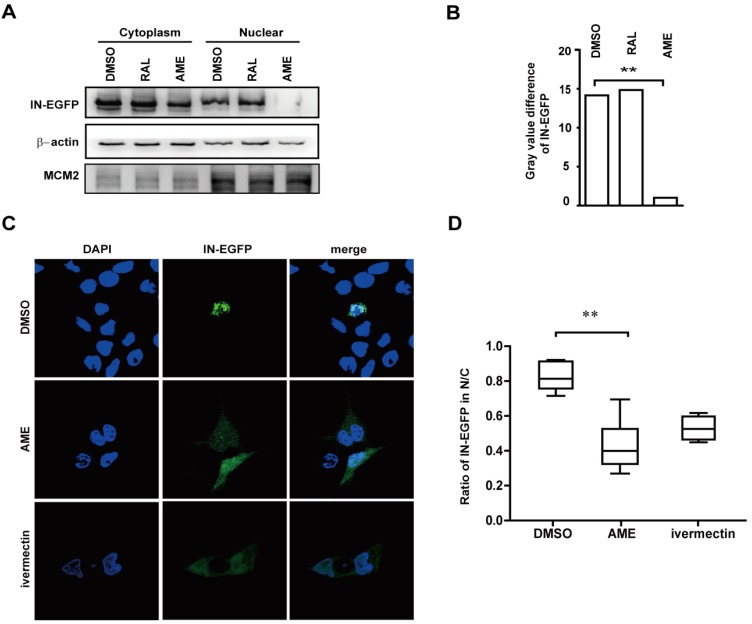
AME directly blocks HIV-1 integrase nuclear entry. (**A**) HEK293T cells were transfected with IN-EGFP. Cells were lysed, and the nucleus and cytoplasm were isolated 40 h post-transfection. IN expression, β-actin, and MCM-2 in nucleus (N) and cytoplasm (C) were measured by Western blotting (left panel); (**B**) The gray value of each band in (A) was quantified and analyzed by ImageJ (right panel); (**C**) HEK293T cells were transfected with IN-EGFP in the presence of DMSO, ivermectin, or AME. Cells were fixed, and the nuclei were stained with 4′6′-diamidino-2-phenylindole (DAPI) after 24 h. Cells were analyzed by confocal microscopy (with a 60× objective lens); (**D**) Ratios of fluorescence in nucleus and whole cells at 488 nm were measured from more than 25 cells per condition randomly selected from three independent experiments. The fluorescence density at 488 nm was quantified by ImageJ and statistical analysis was performed in Graphpad Prism 5.0 software. The data represent the percentage of IN-EGFP in the nucleus. Error bars represent standard deviation (SD) of all cells in each group. ** *p* < 0.01 (determined by Student’s *t* test).

We further tested whether AME directly targets IN rather than other viral components in PIC [16]. To this end, HEK293T cells were transfected with a plasmid expressing IN-EGFP, and then treated with DMSO, RAL, and AME. Levels of IN-EGFP in the nucleus and the cytoplasm were assessed by cytoplasma-nucleus separation followed by Western blotting. A successful isolation of the nucleus and cytoplasm was corroborated by Western blotting of β-actin and MCM-2 in its corresponding lysates. The result showed that IN-EGFP was predominantly localized in the cytoplasm in AME-treated cells as compared with RAL-treated and control cells (Figure 4A). AME inhibits the nuclear entry of IN-EGFP by 14-fold according to gray value of each band quantified by ImageJ (Figure 4B). These data were corroborated by the results of fluorescence imaging (Figure 4C). Like an importin α/β inhibitor ivermectin, AME caused the diffusion of IN into the cytoplasm compared with the diffusion caused by DMSO-treated cells (Figure 4C). Together, these data demonstrated that AME blocked PIC transport into the nucleus by directly by targeting the import of HIV integrase.

**Figure 5:**
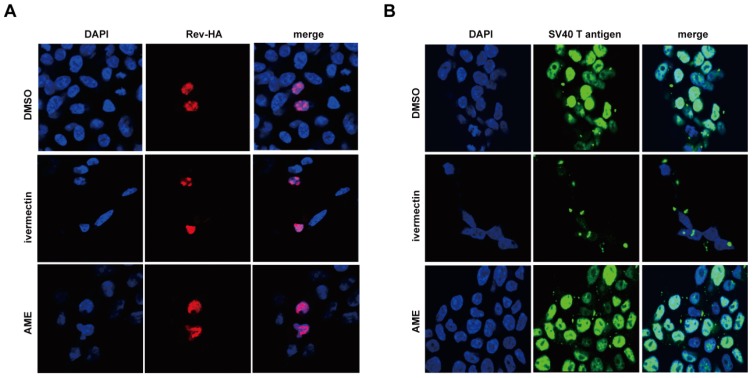
AME does not disturb the classical nuclear transport pathway. (**A**) HeLa cells were transfected with Rev-HA and treated with DMSO, ivermectin or AME. One day post-infection, cells were fixed and stained with mouse anti-HA antibody followed by TRITC-conjugated goat anti-mouse secondary antibody. The nuclei were stained with 4′6′-diamidino-2-phenylindole (DAPI). Cells were analyzed by confocal microscopy (with a 60× objective lens); (**B**) HEK293T cells were treated with DMSO, ivermectin, or AME. One day post-transfection, cells were fixed and stained with mouse anti-SV40 T antigen antibody followed by FITC-conjugated goat anti-mouse secondary antibody, and the nuclei were stained with 4′6′-diamidino-2-phenylindole (DAPI). Cells were examined by confocal microscopy. More than 25 cells per condition spanning three independent experiments were included for each panel.

We next asked whether AME specifically inhibited the nuclear import of PIC, or exerted a general effect on nuclear transport. Nuclear import of cellular proteins mostly depends on nuclear import localization signals (NLS). Classic NLS comprise either a short stretch of basic amino acids, such as SV40 large T antigen (PKKKRKV) or a bipartite NLS including two interdependent stretches of basic amino acids with a spacer cluster in between, such as nucleoplasmin (KRPAATKKAGQAKKKK). Karyphilic proteins containing the classic NLS require both importin α and β for their nuclear entry. Alternatively, other proteins such as Rev, cyclin B1, and hTAP only require importin β. These proteins have arginine rather than lysine in the NLS. In this context, we assessed the effect of AME upon the nuclear transportation of SV40 large T antigen and Rev that are imported into the nucleus by the importin α/β and importin β-mediated pathway, respectively. HeLa cells were transfected with plasmids expressing either HIV-1 Rev or SV40 large T antigen, and treated with DMSO, ivermectin, or AME. Immunofluorescence analysis revealed that AME had no effect upon the nuclear localization of Rev (Figure 5A) or SV40 large T antigen (Figure 5B). In contrast, ivermectin caused cytoplasmic accumulation of SV40 large T antigen, confirming the experimental system is sound (Figure 5B), whereas, HIV-1 Rev, which is dependent on import β alone during nuclear import, was not affected (Figure 5A). These data suggest that AME specifically inhibits the nuclear import of HIV-1 IN without interrupting cellular importin α/β and importin β-mediated nuclear transport pathways.

### 3.4. AME Inhibits the Infection of Different HIV-1 Strains

**Figure 6:**
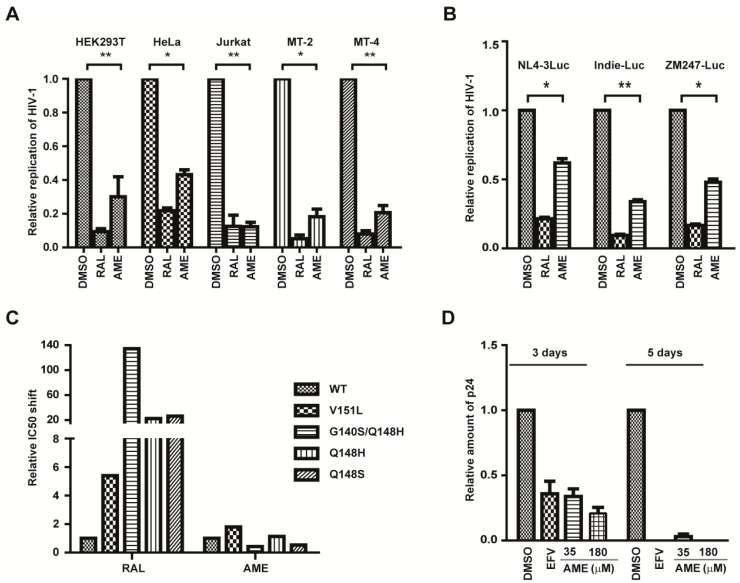
AME inhibits the infection of different HIV-1 strains in different cell lines. (**A**) 5 × 10^5^/mL Jurkat T, MT-2, MT-4, HeLa, and HEK293T cells were infected with VSV-G pseudotyped NL4-3Luc.R-E- viruses in the presence of DMSO, 25 nM RAL, and 60 µM AME. Forty-eight h post-infection, cells were harvested to measure luciferase activity; (**B**) SupT1 cells were infected with VSV-G pseudotyped subtype B NL4-3.luc.R-E- viruses, subtype C ZM247Fv1-Luc, or Indie-C1-Luc viruses treated with 12.5 nM RAL, 35 µM AME, or DMSO as the vehicle control. Two days post-infection, cells were harvested to measure luciferase activity; (**C**) SupT1 cells were infected with VSV-G pseudotyped NL4-3Luc.R-E- viruses or indicated RAL-resistant strains treated with AME, RAL, or DMSO at different concentrations. Cells were harvested to measure luciferase reporter activity. The IC_50_ for WT virus and RAL-resistant mutant virus was calculated; (**D**) Peripheral blood mononuclear cells (PBMCs) were infected with wild type NL4-3 HIV viruses treated with 2 nM EFV, 35 µM or 180 µM AME, or DMSO. Supernatant were harvested to measure the quantity of p24 antigen by ELISA at day 3 and day 5. Data represent the mean ± SD of three independent experiments. * *p* < 0.05, ** *p* < 0.01.

Next, we further evaluated the anti-HIV-1 activity of AME in different cell lines. HEK293T, HeLa, Jurkat T, MT-2, and MT-4 cells were infected with HIV-Luc reporter viruses and then were treated with DMSO, RAL, or AME. AME inhibited HIV-1 infection by approximately 70%, 57%, 88%, 82%, and 80% in HEK293T, HeLa, Jurkat T, MT-2, and MT-4 cells, respectively (Figure 6A). Secondly, we tested the sensitivity of different HIV-1 strains to AME. To this end, we constructed two subtype C chimera viruses, ZM247Fv1Luc (founder/transmitted reporter virus) and Indie-C1-Luc (a primary isolate), and used these viruses to infect SupT1 cells in the presence of DMSO, RAL, or AME. The results showed that AME profoundly inhibited both viruses, with the inhibition rate of approximately 70% and 52% for Indie-C1-Luc and ZM247Fv1Luc, respectively (Figure 6B). Collectively, these data demonstrated that AME inhibits HIV of different origins in a variety of cell lines. Thirdly, we tested the activity of AME against different HIV-1 strains bearing the RAL-resistant mutations including V151L, G140SQ148H, Q148H, and Q148S. As expected, V151L, G140SQ148H, Q148H, and Q148S mutation conferred 5, 134, 23, and 26-fold resistance to RAL, respectively, yet these viruses were inhibited by AME as much as the wild type virus (Figure 6C). With respect to G140SQ148H and Q148S, they exhibit even more susceptibility to AME treatment than wild type.

Suppression of WT HIV replication by AME was also assessed in PBMCs. AME inhibited HIV replication by 66% and 75% at the concentrations of 35 and 180 µM, respectively, at day 3. The anti-HIV activity was more remarkable at day 5, with the inhibition rate of 95% and 100% at concentrations of 35 and 180 µM, respectively (Figure 6D). These data collectively demonstrate that AME inhibits different HIV-1 strains in different cell types and that the RAL-resistant HIV-1 is subject to AME inhibition.

## 4. Discussion

In the present study, we reported for the first time that AME, a natural product from endophytic fungus *Colletotrichum* sp., inhibits HIV-1 infection by targeting the nuclear import of viral integrase. Further studies revealed AME specifically blocked nuclear import of PIC and IN, rather than exerted a general effect on the importin α/β and importin β-mediated nuclear import. Furthermore, AME inhibited both wild type HIV-1 and RAL-resistant strains. These data collectively demonstrate the potential of AME or its derivatives for future development into a novel class of anti-HIV drugs: IN nuclear import inhibitors (INNIIs).

AME significantly reduced the nuclear accumulation of IN but not the SV40 large T antigen and Rev in living cells, indicating that AME selectively inhibits the nuclear import of IN, rather than the importin α/β and importin β-mediated pathway in general. Furthermore, AME exhibited a similar inhibitory effect on the nuclear localization of IN that was either over-expressed alone or as a part of PIC formed in the HIV-1 infected cells. This strongly suggests that AME inhibits HIV-1 replication by targeting either IN and/or IN-specific pathways. Despite that, the detailed mechanism of AME action remains to be elucidated; one possibility is that AME may target the interaction of IN with host factors that are required for nuclear transport. Several lines of evidence indicate that importin α/β are utilized in HIV PIC nuclear transport. To date, two members of importin α, Impα1 and Imp3, and two members of importin β, Imp7 and TRN-SR2, have been shown to interact with HIV-1 IN, while their precise roles in IN nuclear import remain unclear. It is conceivable that AME may bind to either HIV-1 IN or one of these adaptors or receptors and interrupts the interaction of IN with these key host factors. Future studies on the action of AME will provide insight into the detailed mechanism underlying nuclear import of HIV-1 PIC.

Early IN I drug design and discovery has mainly focused on the direct inhibition of enzyme catalytic activities, leading to the development of the first and second generation INSTIs. However, cross-resistance between INSTIs represents a major obstacle. HIV-1 PIC nuclear import inhibitors, such as AME shown in this study, do inhibit INSTI-resistant HIV-1, and, therefore, represent an important class of new anti-HIV drug with a novel mechanism of action. Furthermore, AME effectively inhibits the replication of different HIV-1 strains in several cell lines, suggesting a relatively broad anti-HIV activity of AME. More importantly, AME similarly inhibits both wild-type HIV-1 and RAL-resistant HIV-1, supporting the possible use of IN nuclear import inhibitors to control the infection of INSTI-resistant viruses. Taken together, these data demonstrate the potential of AME for the future development into a novel class of anti-HIV drug. 

In summary, our data demonstrate that AME restricts HIV infection through directly targeting the nuclear import of HIV integrase. This inhibitory effect is highly specific and does not affect the canonical nuclear transport pathway in the host. Our study provides a novel strategy for developing new anti-HIV drugs.
